# Development of a Chemically Defined Medium and Discovery of New Mitogenic Growth Factors for Mouse Hepatocytes: Mitogenic Effects of FGF1/2 and PDGF

**DOI:** 10.1371/journal.pone.0095487

**Published:** 2014-04-17

**Authors:** William C. Bowen, Amantha W. Michalopoulos, Anne Orr, Michael Q. Ding, Donna B. Stolz, George K. Michalopoulos

**Affiliations:** 1 Department of Pathology, University of Pittsburgh School of Medicine, Pittsburgh, Pennsylvania, United States of America; 2 Department of Cell Biology, University of Pittsburgh School of Medicine, Pittsburgh, Pennsylvania, United States of America; IDI, Istituto Dermopatico dell’Immacolata, Italy

## Abstract

Chemically defined serum-free media for rat hepatocytes have been useful in identifying EGFR ligands and HGF/MET signaling as direct mitogenic factors for rat hepatocytes. The absence of such media for mouse hepatocytes has prevented screening for discovery of such mitogens for mouse hepatocytes. We present results obtained by designing such a chemically defined medium for mouse hepatocytes and demonstrate that in addition to EGFR ligands and HGF, the growth factors FGF1 and FGF2 are also important mitogenic factors for mouse hepatocytes. Smaller mitogenic response was also noticed for PDGF AB. Mouse hepatocytes are more likely to enter into spontaneous proliferation in primary culture due to activation of cell cycle pathways resulting from collagenase perfusion. These results demonstrate unanticipated fundamental differences in growth biology of hepatocytes between the two rodent species.

## Introduction

Primary cultures of rodent and human hepatocytes have been used as tools for studies of metabolism, drug toxicity, bile acid synthesis, and many other aspects of hepatic physiology. In addition, growth of rat hepatocytes in primary culture and in chemically defined (serum-free) media has been a very useful tool for determining growth factors (Hepatocyte Growth Factor (HGF), and Epidermal Growth Factor Receptor (EGFR) ligands) that are direct mitogens for rat hepatocytes [1]. These studies paved the progress made for determining the role of EGFR and MET (the HGF receptor) receptors and their ligands in liver regeneration and hepatocyte growth [2]–[5]. While the conditions for hepatocyte growth in chemically defined media have been well defined for the rat [1], definition of similar conditions for mouse hepatocytes has been elusive. Response of mouse hepatocytes to established mitogens such as HGF and EGF in most media has been mediocre at best compared to rat hepatocytes.

The work presented in this manuscript describes a chemically defined serum-free medium that allows growth of mouse hepatocytes in primary culture and high response to growth factors comparable to those seen with the rat. The medium was substantially modified from the hepatocyte growth medium (HGM) used for rat hepatocytes [1]. During the course of thee studies, we also determined that whereas EGFR ligands and HGF are the major direct mitogens for rat hepatocytes, Fibroblast Growth Factors 1 and 2 (FGF1, FGF2) are equally strong direct mitogens for mouse hepatocytes. Platelet Derived Growth Factor (PDGF) also has a weaker direct mitogenic effect. These findings present hitherto undiscovered differences in growth biology of hepatocytes between the two rodent species most commonly used for studies of liver regeneration and carcinogenesis. This work needs to be extended to human hepatocytes. Our work also describes technical difficulties in applying common methodologies used to assess cell proliferation in culture.

## Materials and Methods

### Ethics Statement

Animals (mice) were housed and treated according to institutional guidelines of the University of Pittsburgh IACUC committee (Protocol 1105844). This follows guidelines established by NIH for ethical use of animals in biomedical research. The IACUC committee and the aforementioned protocol specifically approved this study.

Male Fisher 344 rats (2–3 months of age) were purchased from Harlan. Male FVB mice (2–3 months of age) were purchased from Charles River. Animals were allowed food and water ad libitum and handled according to the Ethics Statement above.

### Methods for Alleviation of Animal Suffering

Mice and rats were used to isolate hepatocytes by perfusion of the liver with collagenase. All animals were deeply anesthetized by injection of Nembutal. Surgical exposure of the liver for collagenase perfusion was performed only after the animals were completely anesthetized.

### Isolation and Culture of Hepatocytes

Hepatocytes were isolated by adaptation of the calcium two-step collagenase perfusion technique as previously described [6]. Hepatocytes were plated on collagen-coated six-well plates (BD Biosciences, San Jose, CA) at 250,000 cells/well. After the initial 2-hour attachment period, the medium was changed to formulations as described in Results. There was no serum added at any time during or after plating of hepatocytes. The various growth factors, for either mouse or rat hepatocytes, were added at 24 hours after plating. Media were changed every 48 hours, except for the first medium change, which was done at 24 hours after plating and was used to introduce the added growth factors. Cells were harvested for assessment of cell proliferation at the times indicated in the relevant figures.

### Formulation of the HGM (Hepatocyte Growth Medium) and MHGM (Mouse Hepatocyte Growth Medium)

The formulation of HGM medium has been previously described and was the basis of modifications for the formulation of the MHGM medium.

#### HGM medium

DMEM (Dulbecco’s minimal essential medium), Hepes, glutamine, and antibiotics were purchased from GIBCO/BRL (Gaithersburg, MD). ITS mixture (Insulin, Transferrin, Selenium) was purchased from Boehringer Mannheim. All other additives were cell culture grade (Sigma). Unless otherwise indicated for specific experiments, the basal HGM medium consisted of DMEM supplemented with: purified bovine albumin 2.0 gm/L, glucose 2.25 gr/L, galactose 2.0 gr/L, ornithine 0.1 gm/L, proline 0.030 gm/L, nicotinamide 0.610 gm/L, ZnCI_2_ 0.544 mg/L, ZnSO_4_∶7H2O 0.750 mg/L, CuSO_4_∶5H_2_O 0.20 mg/L, MnSO_4_ 0.025 mg/L, glutamine 5.0 mM, ITS 1.0 gm/L, (rh-insulin 5.0 mg/L, human transferrin 5.0 mg/L [30% diferric iron saturated], selenium 5.0 µg/L), and dexamethasone 10^−7^ M. Penicillin and streptomycin were added to the basal HGM at 100 mg/L each. The mixed basal HGM was sterilized by filtration through a 0.22-µm low protein-binding filter system, stored at 4°C, and used within 4 wk. The growth factors, as required, were added to medium fresh at the specified concentrations every time the medium was changed.

#### MHGM medium

MHGM was derived by using E-MEM (from Sigma) instead of DMEM as the basis for the formulation. Nicotinamide was omitted and dexamethasone was added at a final concentration of 10^−6^ M (ten times more than HGM). Glucose concentration for MHGM 4.5 mg/ml) was also adjusted to two-fold that of HGM.

### Hepatocyte Proliferation Assay

**Measurement of DNA synthesis.**

#### 1. Thymidine incorporation

H^3^]Thymidine was added to the medium at 24 hours at a concentration of 2.5 µCi/ml. The medium was removed when the cultures were harvested and hepatocytes were fixed with ice-cold 5% trichloroacetic acid. Trichloroacetic acid was removed and the plates were washed in running tap water and air-dried completely. Then 750 µL 0.33 M NaOH was added to each well for 30 minutes to solubilize the cells. The solution was transferred to a new tube and 250 µL of 40% TCA/1.2 M HCl was added for precipitation. The tubes were centrifuged at 12,000 g for 10 minutes and the pellets were dissolved in 500 µL 0.33 M NaOH. A 200-µL aliquot was used to measure Η^3^ cpm in a Beckman LS6000IC scintillation counter (Beckman Coulter, CA) and 100 µL was used to determine optical density value of total DNA. Data are plotted as CPM/µg DNA. BRDU incorporation into DNA was assessed by the use of the Invitrogen BRDU staining kit (from Life Technologies).

#### 2. Bromodeoxyuridine (BRDU) and Ki67 immunohistochemistry

Ki-67 antibodies were obtained from Santa Cruz. BRDU immunohistochemistry was performed using the specific kit from Becton-Dickinson.

#### Concentrations of mitogenic growth factors

We performed dose-response studies (data not shown) and assessed that 40 ng/per ml exceeded the highest dose response for each growth factor. They were all added to the medium at 40 ng/ml to assure maximal response.

#### Transmission electron microscopy

Hepatocytes grown on tissue culture collagen coated plates were fixed in 2.5% glutaraldehyde in 100 mM PBS (8 gm/l NaCl, 0.2 gm/l KCl, 1.15 gm/l Na_2_HPO_4_^.^7H_2_O, 0.2 gm/l KH_2_PO_4,_ pH 7.4 ) overnight at 4°C. Hepatocyte monolayers were then washed in PBS three times then post-fixed in aqueous 1% osmium tetroxide, 1% Fe_6_CN_3_ for 1 hr. Cells were washed 3 times in PBS then dehydrated through a 30–100% ethanol series then several changes of Polybed 812 embedding resin (Polysciences, Warrington, PA). Cultures were embedded in by inverting Polybed 812-filled BEEM capsules on top of the cells. Blocks were cured overnight at 37°C, then cured for two days at 65°C. Monolayers were pulled off the coverslips and re-embedded for cross section. Ultrathin cross sections (60 nm) of the cells were obtained on a Riechart Ultracut E microtome, post-stained in 4% uranyl acetate for 10 min and 1% lead citrate for 7 min. Sections were viewed on a JEOL JEM 1011 transmission electron microscope (JEOL, Peobody MA) at 80 KV. Images were taken using a side-mount AMT 2 k digital camera (Advanced Microscopy Techniques, Danvers, MA).

## Results

### Formulation of MHGM Medium for Mouse Hepatocytes

Our initial studies with mouse hepatocytes in the rat-defined HGM medium showed poor responsiveness to growth factors as well as substantial “spontaneous” growth of mouse hepatocytes without serum or any growth factor addition. We have shown in previous studies in vivo that remodeling of liver extracellular matrix at the early stages of liver regeneration is associated with release and activation of HGF [2]. We have also found that pre-treatment of the liver of live rats with collagenase dramatically enhanced the mitogenic effects of exogenously administered HGF [7]. We hypothesized that collagenase perfusion of the mouse liver to isolate hepatocytes causes disruption of the extracellular matrix (the very purpose of collagenase perfusion) and results in activation of cell-cycle driving cascade signaling, perhaps triggered by the release of matrix-bound HGF. Spontaneous entry of hepatocytes into cell cycle following isolation by collagenase perfusion has been previously shown for the rat [8], [9]. However, rat hepatocytes do not enter into DNA synthesis in the absence of growth factors and there is no high rate of “spontaneous” proliferation of rat hepatocytes in primary culture. Mouse hepatocytes on the other hand have highly variable rates in that regard, with BRDU labeling indices as high as 30%–40% in the first and second day in primary culture (data not shown) with substantial variation seen from one hepatocyte isolate to another. This rate of proliferation subsides by Day 2. Thus we proceeded to conduct our cell proliferation studies with mouse hepatocytes after 24 hours in primary culture and maintained mouse hepatocytes in plain medium without any addition of serum from the time of plating to the time of harvesting.

**Figure 1:**
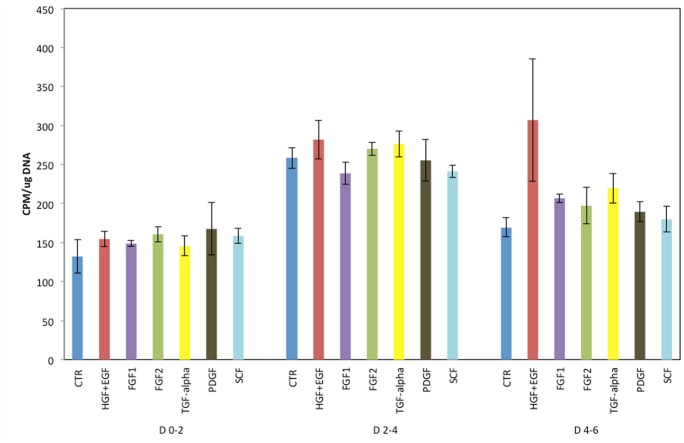
Proliferation of mouse hepatocytes maintained in the rat hepatocyte (HGM) medium. Y-axis indicates incorporation of tritiated thymidine per µg DNA. The growth factors added to the medium are listed under the X-axis. CTR: Control cultures, with no growth factors added. Growth factors were added at 24 hours after plating of hepatocytes. D 0–2 (etc.) indicates days in culture after addition of the growth factors. (For growth factor concentration, please see Materials and Methods).

**Figure 2:**
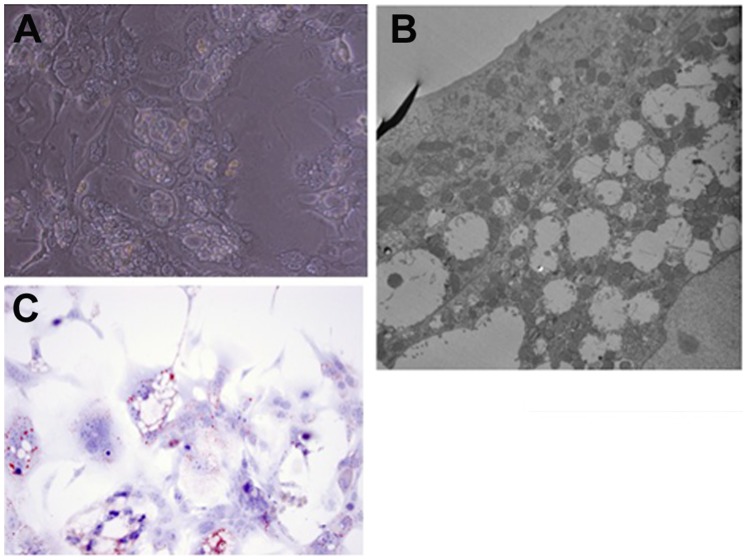
Intense vacuolation of hepatocyte cytoplasm in a preliminary formulation of the mouse hepatocyte growth medium (MHGM). Elimination of nicotinamide removed this cytoplasmic change. A: Phase contrast photomicrograph of the hepatocytes in culture showing intense valuation of the cytoplasm (Magnification: 100X). B: Electron micrograph (5,000X) of hepatocytes carrying vacuoles. There is no apparent connection with bile canaliculi or autophagosomes. C: Oil Red-O stain for lipid demonstrates that the vacuoles observed did not contain lipids.

We also observed that even starting from 24 hours in culture, mouse hepatocytes kept in (rat-defined) HGM medium had poor response to growth factors, as shown in Figure 1. A better response was seen when the basic formulation of the mouse medium (to be referred as MHGM) was changed from the HGM basal medium of DMEM to Eagle’s MEM (EMEM). We did observe however that addition of HGF and EGF into that medium, while having minimal effect, was causing substantial vacuolation of the cytoplasm of the mouse hepatocytes (Figures 2 A and B). Vacuoles did not contain triglycerides, as evidence by an Oil Red-O stain (Figure 2 C). The vacuoles were present within the cytoplasm of hepatocytes and did not represent dilated bile canaliculi or autophagic vacuoles. The vacuoles did not appear when nicotinamide was removed from the medium. It should be noted that no such effect with nicotinamide was observed with rat hepatocytes and, to the contrary, presence of nicotinamide was essential for rat hepatocyte proliferation [1].

**Figure 3:**
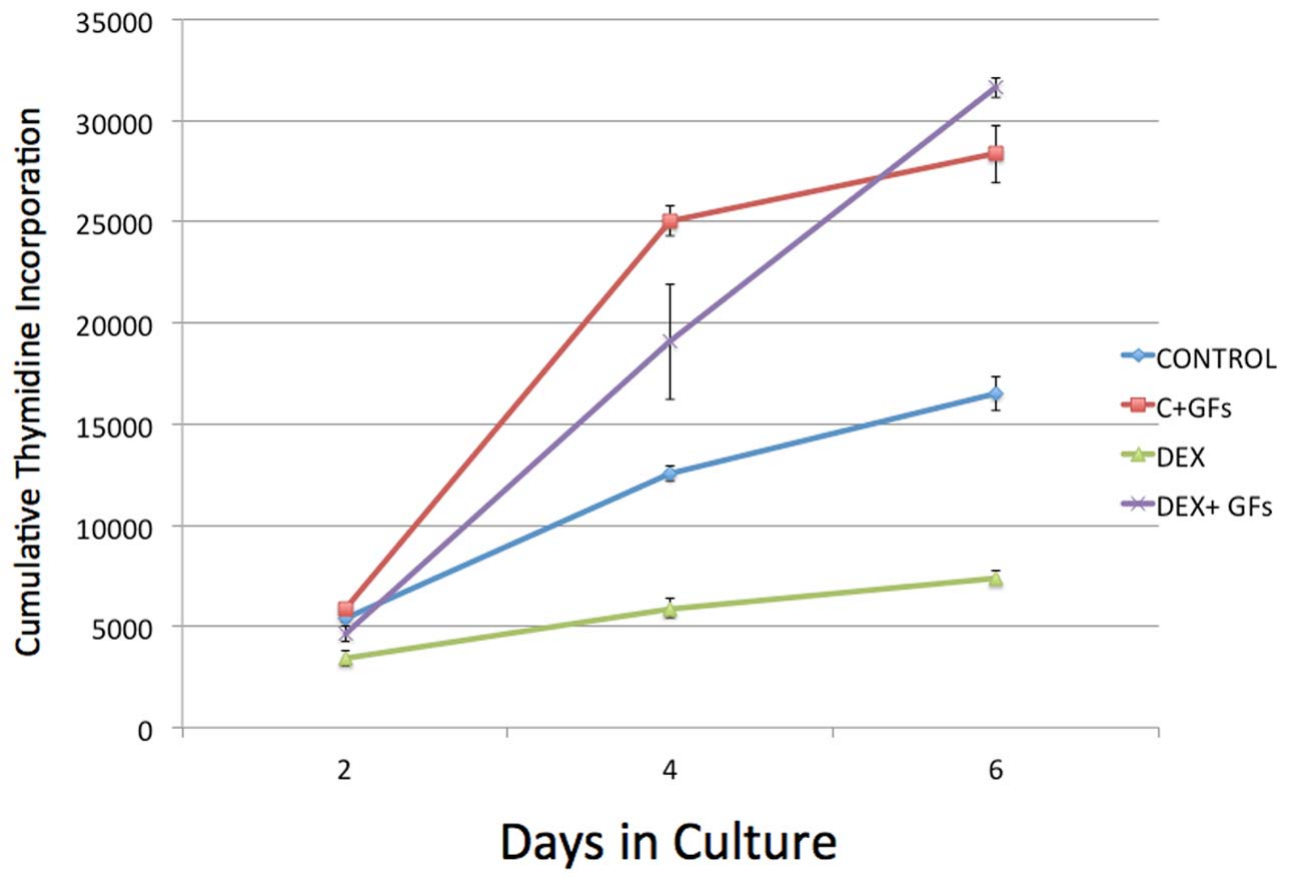
Suppression of spontaneous proliferation of mouse hepatocytes in the presence of 10 ^−**6**^ **M dexamethasone.** Tritiated thymidine was added continually in the cultures, from 4 hours after cell plating following perfusion of the liver by collagenase. Cultures were harvested at the indicated time points in the X-axis. Addition of HGF and EGF is indicated as “GFs”. (For growth factor concentration, please see Materials and Methods).

Another important factor for the MHGM media formulation was the concentration of the steroid dexamethasone. Increasing dexamethasone concentration from 10^−7^ M (in the HGM) to 10^−6^ M in the MHGM medium substantially suppressed the spontaneous proliferation rate of mouse hepatocytes without affecting the response to growth factors (Figure 3).

The detail final formulation and comparison of the ingredients between the rat hepatocyte-defined HGM medium and the mouse hepatocyte-optimized MHGM medium are described in Materials and Methods.

### Growth Factors Stimulating Proliferation of Mouse Hepatocytes in the MHGM Medium

**Figure 4:**
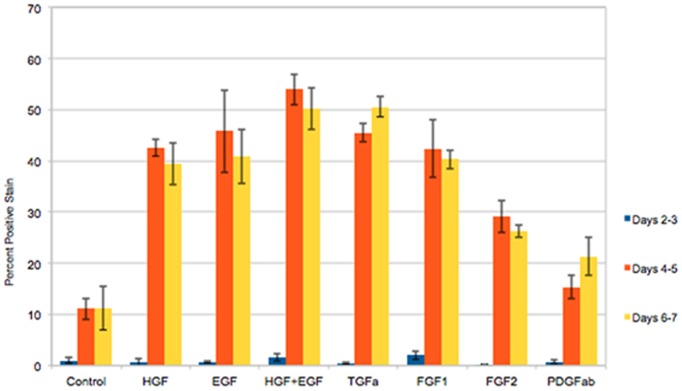
Mouse hepatocyte proliferation in the final formulation of the MHGM medium. Y-axis indicates percent of labeled nuclei due to incorporation of bromodeoxyuridine into DNA. The growth factors added to the medium are listed under the X-axis. CTR: Control cultures, with no growth factors added. Growth factors were added at 24 hours after plating of hepatocytes. D 0–2 (etc.) indicates days in culture after addition of the growth factors. (For growth factor concentration, please see Materials and Methods).

Proliferation of mouse hepatocytes exposed to different growth factors in the MGM medium is shown in Figure 4. Hepatocytes were exposed to 2-day pulses of BRDU at the indicated days and the nuclear labeling index of the cells was assessed. The results show hepatocyte proliferation rates in response to EGF, HGF, (EGF plus HGF), and TGFα, primarily at days 4–7 in culture. Minimal spontaneous proliferation was seen in control cultures without growth factor addition. We also noticed that FGF1 and FGF2 had a strong mitogenic effect, something not seen with rat hepatocytes. Measurable mitogenic effect was also seen with PDGF AB. Immunohistochemical stains for desmin showed that the small effect of PDGF AB on cell proliferation was on desmin-negative cells (hepatocytes) and not on contaminant (desmin positive) stellate cells. There was no further stimulation of hepatocyte proliferation by addition of higher concentrations of PDGF (data not shown).

It should be noted that the results of these studies were obtained by two-day BRDU pulses. When, instead of BRDU, we used immunohistochemistry for the cell cycle specific nuclear protein Ki67, most of the cells appearred positive, whether they were in control or growth factor-exposed cultures. We consider the increased expression of Ki67 as part of the overall change in cell cycle related protein expression induced by the collagenase perfusion and the culture itself. Expression of cell cycle genes (but not DNA synthesis) has been observed in the past with rat hepatocytes [9], [10]. Since BRDU clearly reflects DNA synhesis, we considered that as the method of choice to measure the effect of growth factors on mouse hepatocyte proliferation.

### Rat Hepatocytes in the Mouse Medium Perform as Expected and Reflect Species Differences

**Figure 5:**
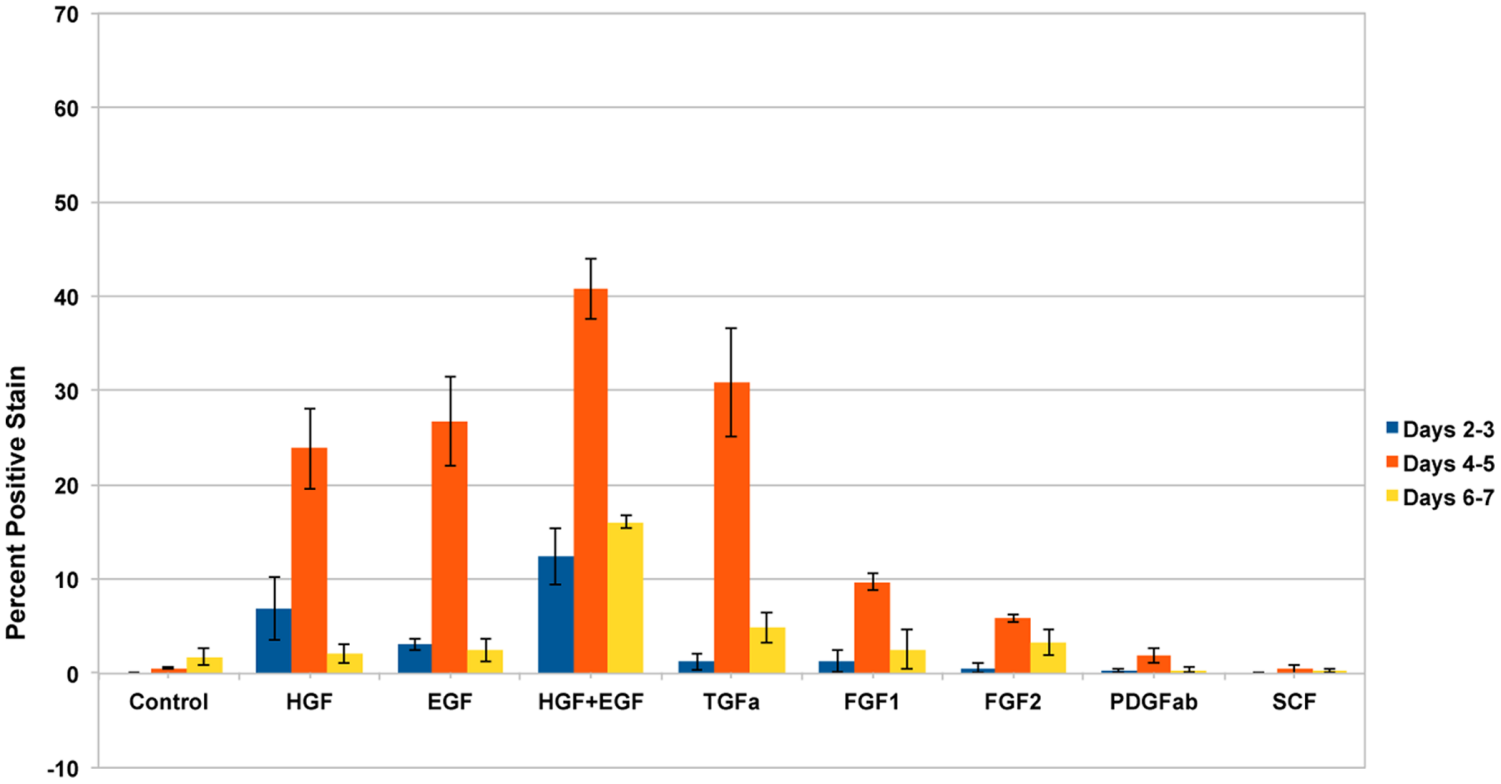
Rat hepatocyte proliferation in the MHGM medium. Y-axis indicates percent of labeled nuclei due to incorporation of bromodeoxyuridine into DNA. The scale of Y-axis is kept identical to that of figure 4, for direct comparison of the responses of mouse and rat hepatocytes. The growth factors added to the medium are listed under the X-axis. CTR: Control cultures, with no growth factors added. Growth factors were added at 24 hours after plating of hepatocytes. D 0–2 (etc.) indicates days in culture after addition of the growth factors. (For growth factor concentration, please see Materials and Methods).

We were concerned that the mitogenic effects of FGF1/2 and PDGF AB on mouse hepatocytes might have been missed with rat hepatocytes, due to differences in the cell culture medium. To that end, we assessed proliferation of rat hepatocytes in the MHGM medium, in exactly the same conditions as with the mouse hepatocytes. The results are shown in Figure 5. Though overall proliferation rates were lower than those seen in the HGM medium, rat hepatocytes responded as previously described, with very little proliferation in response to FGF1 and much less to FGF2. No substantial proliferation was induced by PDGF AB. The lower proliferations rates of rat hepatocytes in MHGM may be due to the absence of nicotinamide and the higher concentration of dexamethasone, both of which are likely to negatively affect rat hepatocyte proliferation, as we have previously described [1].

### Interactions between FGF2 and PDGF

**Figure 6:**
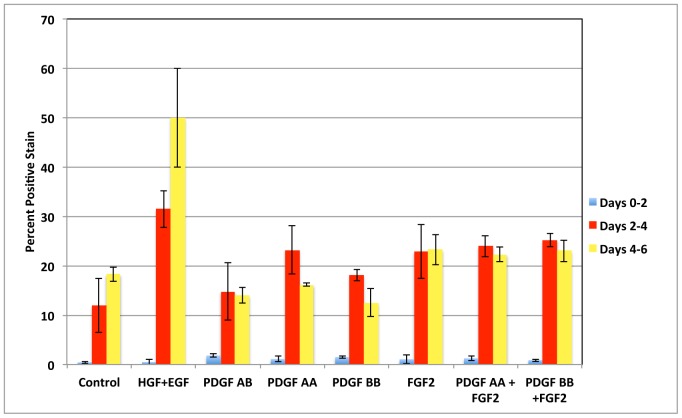
Interactions between FGF2 and PDGF AA and BB in stimulation of DNA synthesis in mouse hepatocyte cultures. Y-axis indicates percent of labeled nuclei due to incorporation of bromodeoxyuridine into DNA. The growth factors added to the medium are listed under the X-axis.

Previous studies have shown that in several cell types there is interaction between PDGF BB and FGF2, as a result of which FGF2 is often found to inhibit the effects of PDGF BB and vice versa [11]–[13](see Discussion). In order to investigate such interaction between FGF2 and PDGF, we explored the rate of DNA synthesis (assessed by BRDU incorporation) in cultures of mouse hepatocytes kept in the chemically defined MHGM medium, and exposed to PDGF AA, PDGF BB in the presence or absence of FGF2 (Fig. 6). Our results do not show negative interactions between FGF2 and PDGF AA or BB in terms of stimulation of DNA synthesis.

## Discussion

Our findings demonstrate that there are fundamental differences in response to growth factors between mouse and rat hepatocytes in culture, probably as a result of different responses to degradation of extracellular matrix and release of bound growth factors and/or integrin signaling via collagenase perfusion. Our findings also demonstrate that the family of fibroblast growth factors (FGF) plays a more important role in mouse hepatocyte growth biology.

Previous studies have demonstrated that FGF1 and FGF2 may contribute to hepatocyte growth. Detail studies have shown that hepatocytes express the fibroblast growth factor receptor FGFR4 but not the receptors FGFR1 and FGFR2 [14]. The effects of FGFR4 are dependent on association with the transmembrane protein βklotho [15]. This protein is often deleted in hepatocellular carcinomas and the results suggest that the interaction of βklotho and FGFR4 suppress proliferation of neoplastic hepatocytes. FGFR1 and FGFR2 are expressed in other hepatic cell types (endothelial and stellate cells) [16]. FGF1 and FGF2 expression by hepatocytes increases during liver regeneration [17]. The increased expression occurs after hepatocytes have already committed to enter into S phase. Genetic deletion of FGFR4 has been associated with defects in cholesterol and bile acid metabolism but there were no demonstrable effects on hepatocyte proliferation in response to injury [14]. In another study, however, hepatocyte-targeted expression of a dominant negative FGFR2 suppressed and delayed liver regeneration [18]. FGF1 and FGF2 have been associated with induction of the hepatic primordium from the foregut endoderm [19]. However, mice with combined deletion of FGF1 and FGF2 have normal embryonic development, suggesting that the absence of FGF1 and FGF2 is compensated by other members of the 23-member family of FGFs, which use the same receptors as FGF1 and FGF2 [16]. Combined FGF1/FGF2 knockout mice do show increased hepatocyte sensitivity to CCl4 but have a decreased level of stellate cell-derived matrix collagen type 1 synthesis in response to long term CCl4 exposure [16]. Recent studies have also focused on mouse FGF15, the ortholog of human FGF19. Expression of these FGFs is induced by bile acids on enterocytes of the ileum. Migration to the liver has endocrine effects on hepatocyte-mediated cholesterol and bile acid metabolism. Mice with systemic genetic elimination of FGF15 had delayed liver regeneration [20]. Our findings and the studies cited above suggest that the FGF family members have effects on hepatocyte growth that need to be further studied and understood in the context of liver regeneration and chronic liver injury. The likelihood of important mitogenic effects of FGF members on hepatocytes is further enhanced by a recent analysis of the signal transduction pathways of receptor tyrosine kinases. Based on the inferred signaling networks, that study demonstrated that EGFR, MET and FGFR1 belong to the same subclass in terms of intracellular signaling [21].

Much less evidence from the literature exists on effects of PDGF on hepatocytes. Transgenic over-expression of PDGF C in hepatocytes was associated with increased activation of stellate cells, hepatic fibrosis and steatosis, and development of hepatocellular carcinoma. It was not clear whether the increased HCC development was due to direct effects of PDGF or to increased synthesis of HGF by stellate cells [22]. Hepatocyte targeted genetic elimination of PDGFRα is associated with delay in liver regeneration and compensatory increases in EGFR and MET [23]. There is also increased expression of PDGFRα in mice with hepatocyte-targeted genetic elimination of beta catenin [24]. Given the smaller response to PDGF compared to the other mitogenic growth factors, it is conceivable that there is a specific sub-class of hepatocytes, perhaps related to specific lobular localization, that have the capacity to respond to PDGF.

Several previous studies have indicated that PDGF BB and FGF2 exert negative effects on each other’s activities in several cell types. PDGF BB inhibits angiogenic effects of FGF2 both in vitro and in vivo, and this action is likely mediated by PDGFRα, since neutralization of the latter eliminates the inhibitory effects of PDGF BB [13]. The interaction is complex and involves both the ligands and their receptors. FGF2 and PDGF BB bind directly with each other [11]. In addition, there is strong evidence that FGFR1 and PDGFRα form heterodimers, through which PDGF BB and FGF2 can have very subtle effects on angiogenesis [12]. The absence of effects of PDGF BB on FGF2 stimulated hepatocyte DNA synthesis probably reflects different patterns of receptor interaction. Since FGF2 and PDGF BB bind directly, this cannot be the reason for the observed lack of interaction in our study. Localization of receptors on plasma membrane in hepatocyte cultures, however, is much different than in hepatocytes in intact liver [25]. The change of the hepatocyte shape from cuboidal (in vivo) to a flattened epithelial cell in culture has been found to have complex effects on intermolecular protein associations and other structural changes, including absence of bile canaliculi, etc. Addition of complex matrix material restores such structures bu it also inhibits hepatocyte DNA synthesis, thus not allowing a direct study of the PDGF BB and FGF2 interactions in hepatocytes maintaing proper cell polarity.

In summary, our current study provides information about the conditions required in cell culture for study of mouse hepatocyte growth biology and documents that members of the FGF family and, to a lesser extent, PDGF, should be considered further by in vivo studies for playing a role in liver regenerative responses.
